# Dual Roles for DNA Polymerase Theta in Alternative End-Joining Repair of Double-Strand Breaks in Drosophila

**DOI:** 10.1371/journal.pgen.1001005

**Published:** 2010-07-01

**Authors:** Sze Ham Chan, Amy Marie Yu, Mitch McVey

**Affiliations:** 1 Department of Biology, Tufts University, Medford, Massachusetts, United States of America; 2 Program in Genetics, Tufts Sackler School of Graduate Biomedical Sciences, Boston, Massachusetts, United States of America; Stowers Institute for Medical Research, United States of America

## Abstract

DNA double-strand breaks are repaired by multiple mechanisms that are roughly grouped into the categories of homology-directed repair and non-homologous end joining. End-joining repair can be further classified as either classical non-homologous end joining, which requires DNA ligase 4, or “alternative” end joining, which does not. Alternative end joining has been associated with genomic deletions and translocations, but its molecular mechanism(s) are largely uncharacterized. Here, we report that *Drosophila melanogaster* DNA polymerase theta (pol theta), encoded by the *mus308* gene and previously implicated in DNA interstrand crosslink repair, plays a crucial role in DNA ligase 4-independent alternative end joining. In the absence of pol theta, end joining is impaired and residual repair often creates large deletions flanking the break site. Analysis of break repair junctions from flies with *mus308* separation-of-function alleles suggests that pol theta promotes the use of long microhomologies during alternative end joining and increases the likelihood of complex insertion events. Our results establish pol theta as a key protein in alternative end joining in Drosophila and suggest a potential mechanistic link between alternative end joining and interstrand crosslink repair.

## Author Summary

DNA double-strand breaks, in which both strands of the DNA double helix are cut, must be recognized and accurately repaired in order to promote cell survival and prevent the accumulation of mutations. However, error-prone repair occasionally occurs, even when accurate repair is possible. We have investigated the genetic requirements of an error-prone break-repair mechanism called alternative end joining. We have previously shown that alternative end joining is frequently used in the fruit fly, *Drosophila melanogaster*. Here, we demonstrate that a fruit fly protein named DNA polymerase theta is a key player in this inaccurate repair mechanism. Genetic analysis suggests that polymerase theta may be important for two processes associated with alternative end joining: (1) annealing at short, complementary DNA sequences, and (2) DNA synthesis that creates small insertions at break-repair sites. In the absence of polymerase theta, a backup repair mechanism that frequently results in large chromosome deletions is revealed. Because DNA polymerase theta is highly expressed in many types of human cancers, our findings lay the groundwork for further investigations into how polymerase theta is involved in repair processes that may promote the development of cancer.

## Introduction

DNA double-strand breaks (DSBs) and interstrand crosslinks pose serious threats to cell survival and genome stability. Because these lesions compromise both strands of the double helix, they impede DNA replication and transcription and therefore must be removed in a timely and coordinated manner. Interstrand crosslink repair has been shown to involve a DSB intermediate in some cases (reviewed in [1]). Therefore, there may be substantial mechanistic overlap in the processes used during repair of these two lesions.

Error-free repair of DSBs can be accomplished through homologous recombination (HR) with an undamaged homologous template (reviewed in [2]). However, in contexts where suitable templates for HR do not exist, error-prone repair mechanisms are also used. For example, non-homologous end joining (NHEJ) frequently creates small insertions and deletions during DSB repair, particularly in cases where the broken ends cannot be readily ligated (reviewed in [3]). Analogously, the use of translesion DNA polymerases during interstrand crosslink repair can result in mutations, due to the reduced fidelity of these polymerases [4], [5].

Accumulating evidence suggests that NHEJ is composed of at least two genetically distinct mechanisms. Classical NHEJ (C-NHEJ) involves the sequential recruitment of two highly conserved core complexes (reviewed in [6]). First, the Ku70/80 heterodimer recognizes and binds to DNA ends in a sequence-independent manner, thereby protecting them from degradation. In many eukaryotes, Ku70/80 also recruits DNA-PK_cs_, forming a synaptic complex that can recruit additional processing enzymes such as the Artemis nuclease and the X family DNA polymerases mu and lambda. These proteins expand the spectrum of broken ends that can be rejoined. The second core complex, composed of DNA ligase 4, XRCC4, and XLF/Cernunnos, catalyzes ligation of the processed ends. Depending on the substrate, C-NHEJ can result in perfect repair of broken DNA, or it can result in small deletions of 1–10 nucleotides and/or insertions of 1–3 nucleotides [7]. Although C-NHEJ can repair blunt-ended substrates, a subset of C-NHEJ products appear to involve annealing at 1–4 nucleotide microhomologous sequences on either side of the break.

Alternative end-joining (alt-EJ) is defined as end-joining repair that is observed in cells or organisms lacking one or more C-NHEJ components (reviewed in [8]). Alt-EJ in yeast is associated with deletions larger than those typically created by C-NHEJ, together with an increased tendency to repair by annealing at microhomologous sequences. Ku and ligase 4-independent end joining observed in mammalian cells also displays an increased tendency towards use of short microhomologies compared to C-NHEJ [9], [10]. Therefore, alt-EJ is sometimes called microhomology-mediated end joining (MMEJ) [11]. However, the relationship between MMEJ and alt-EJ is still unclear, and alt-EJ may comprise one or more C-NHEJ-independent repair mechanisms [8].

The importance of alt-EJ repair is highlighted by multiple studies that suggest it may promote chromosome instability and carcinogenesis. Alt-EJ produces chromosome translocations in mouse embryonic stem cells lacking Ku70 [12] and the use of alt-EJ during V(D)J recombination in C-NHEJ-deficient murine lymphocytes causes complex chromosome translocations and progenitor B cell lymphomas [13]. Furthermore, alt-EJ has been implicated in various translocations associated with chronic myeloid leukemia and human bladder cancer [14], [15]. Importantly, alt-EJ also operates during V(D)J rejoining in C-NHEJ-proficient B lymphocytes [16], suggesting that its role in DSB repair is not limited to situations where C-NHEJ is defective. However, alt-EJ is frequently masked by more dominant repair processes that are essential for vertebrate development, making it difficult to study. Therefore, its molecular mechanisms and the proteins involved remain largely unknown.

Several lines of evidence demonstrate that Drosophila is an excellent model system in which to study alt-EJ in a metazoan. The Drosophila genome lacks several mammalian C-NHEJ components, including DNA-PK_cs_ and Artemis. This may predispose flies towards non-C-NHEJ repair. Consistent with this, we have previously shown that a DSB caused by excision of a *P* element transposon in flies is readily repaired by a DNA ligase 4-independent end-joining process [17]. Interestingly, although Drosophila orthologs for the Pol X family DNA polymerases mu and lambda have not been identified [18], we and others have found evidence for polymerase activity in Drosophila end-joining repair [17], [19], [20]. Specifically, end joining in flies is often associated with the insertion of nucleotides at repair junctions, frequently involving imperfect repeats of 5–8 nucleotides. Full or partial templates for the insertions, occasionally possessing mismatches, can often be identified in adjacent sequences, suggesting the action of an error-prone polymerase. Similar templated nucleotides (T-nucleotides) have previously been identified at translocation breakpoints in human lymphomas [21]–[23]. Therefore, T-nucleotides could represent a signature of alt-EJ and may be informative regarding its molecular mechanisms.

Additional insight into alt-EJ is provided by recent reports suggesting a mechanistic link between alt-EJ and interstrand crosslink repair. For example, a study of two Chinese hamster ovary cell lines sensitive to the crosslinking agent mitomycin C found that they were also deficient in alt-EJ [24]. Furthermore, certain interstrand crosslink-sensitive cell lines from Fanconi Anemia patients are also impaired in DNA-PK_cs_-independent rejoining of linearized plasmids [25]. Based on these reports, we hypothesized that additional mechanistic insight into both interstrand crosslink repair and alt-EJ could be gained by searching for mutants defective in both processes. To test this, we have screened Drosophila mutants that are sensitive to DNA crosslinking agents for additional defects in alt-EJ repair. In this work, we describe our studies with one such mutant, *mus308*.

The *mus308* (mutagen sensitive 308) mutant was originally identified by its extreme sensitivity to interstrand crosslinking agents but normal resistance to alkylating agents [26]. Subsequently, *mus308* was found to code for DNA polymerase theta, which is most similar to A family DNA polymerases such as *Escherichia coli* Pol I [27]. Orthologs of polymerase theta (hereafter referred to as pol θ) are found in many metazoans, including *Caenorhabditis elegans*, *Arabidopsis thaliana*, and *Homo sapiens*, but not in unicellular eukaryotes, including the yeasts [28]–[30]. Several lines of evidence suggest that pol θ may play an important role in maintaining genome stability. Similar to flies, *C. elegans* with mutations in *POLQ-1* are defective in repair of interstrand crosslinks [28]. Mice lacking pol θ (*chaos1* mutants) have a high frequency of spontaneous and mitomycin C-induced micronuclei in erythrocytes, consistent with genomic instability [31]. In addition, vertebrate pol θ orthologs have been implicated in a wide range of repair processes, including base excision repair, bypass of abasic sites, and somatic hypermutation of immunoglobulin genes [32]–[36]. Finally, upregulation of pol θ is observed in a variety of human tumors and is associated with a poor clinical outcome, suggesting that its overexpression may contribute to cancer progression [37].

Pol θ is unusual in possessing an N-terminal helicase-like domain and a C-terminal polymerase domain. Although pol θ purified from human cell lines and Drosophila has error-prone polymerase activity and single-stranded DNA-dependent ATPase activity, helicase activity has not been demonstrated *in vitro* [30], [38], [39]. Therefore, it remains unclear exactly how the structure of pol θ relates to its multiple functions in DNA repair in different organisms.

We report here that in addition to its role in DNA interstrand crosslink repair, Drosophila pol θ is involved in end-joining repair of DSBs. This alt-EJ mechanism operates independently of both Rad51-mediated HR and ligase 4-dependent C-NHEJ. Genetic analysis using separation-of-function alleles provides support for distinct roles of both the N- and C-terminal domains of pol θ in alt-EJ. Collectively, our data support a model in which helicase and polymerase activities of Drosophila pol θ cooperate to generate single-stranded microhomologous sequences that are utilized during end alignment in alt-EJ.

## Results

### Drosophila pol θ is important for both interstrand crosslink repair and end-joining repair of DNA double-strand breaks

Drosophila *mus308* mutants were initially identified based on their sensitivity to low doses of chemicals that induce DNA interstrand crosslinks [26]. To confirm this phenotype, we assembled a collection of previously identified *mus308* mutant alleles [40], [41] and measured the ability of hemizygous mutant larvae to survive exposure to the crosslinking agent mechlorethamine (nitrogen mustard). Of the mutants that we tested, four were unable to survive exposure to 0.005% mechlorethamine: *D2*, *D5*, *2003*, and *3294* (data not shown), consistent with their inability to repair interstrand crosslinks.

**Figure 1:**
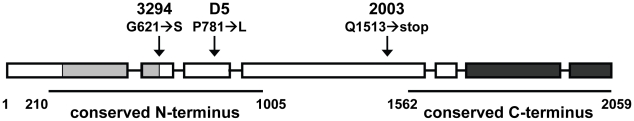
Schematic of the *mus308* gene. Exons are represented by boxes. The helicase-like domain (light gray shading) and polymerase domain (dark gray shading) are shown. Locations of the *D5*, *3294*, and *2003* point mutations are indicated with arrows (numbers correspond to amino acid positions in the protein). Not shown is the *D2* mutation, which results in severely reduced levels of pol θ.

To determine the molecular lesions responsible for mechlorethamine sensitivity, we sequenced the entire *mus308* coding region of flies hemizygous for each mutant allele. Pol θ possesses both a conserved N-terminal helicase-like domain and a C-terminal pol I-like polymerase domain (Figure 1, Figure S1) [30]. Three of the four alleles contain unique sequence changes that are predicted to affect pol θ primary structure (Figure 1, Figure S2, and Figure S3). The *2003* allele is a nonsense mutation upstream of the polymerase domain, while the *D5* allele is a missense mutation that alters a highly conserved proline in the conserved N-terminus. The *3294* allele changes an invariant glycine in the helicase domain to serine. Interestingly, this residue is conserved in the related *mus301* helicase, but not in other DNA helicases (data not shown). No mutations were found in the coding sequence of the *D2* allele. Because homozygous *D2* flies have undetectable levels of pol θ protein [38], the *D2* mutation may affect a regulatory region of *mus308*.

**Figure 2:**
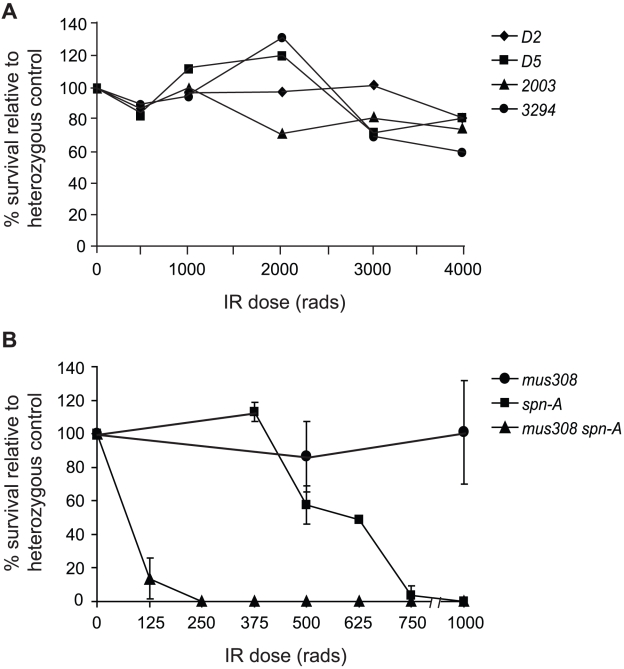
Drosophila pol θ is required for repair of IR–induced damage in the absence of Rad51. (A) *mus308* mutants are not sensitive to ionizing radiation. Female flies heterozygous for the indicated *mus308* alleles were mated to *Df(3R)Sz29*/*TM3* males and third instar larval progeny were irradiated with increasing doses of IR. The percent survival of *mus308* hemizygous mutants was calculated relative to an unirradiated control. Each dose was repeated twice; the average of the two experiments is shown. (B) *spn-A mus308* double mutants are extremely sensitive to ionizing radiation. Heterozygous *spn-A^057^mus308^D5^* females were mated to heterozygous *spn-A^093^mus308^D5^* males and third instar larval progeny were irradiated with increasing doses of IR. The percent survival of *spn-A mus308* compound heterozygotes was calculated relative to an unirradiated control. Each dose was repeated at least twice. Error bars represent standard deviations.

One explanation for the extreme sensitivity of *mus308* mutants to mechlorethamine could be a defect in the repair of certain types of DSB intermediates that are created during crosslink repair. To test this, we exposed flies hemizygous for each mutant allele to increasing doses of ionizing radiation (IR). Although IR creates many different types of lesions, unrepaired DSBs are thought to be the main cause of cell death following irradiation. All four *mus308* mutants survived IR exposures as high as 4000 rads (Figure 2A), although bristle and wing defects characteristic of apoptotic cell death were frequently observed at high doses. Drosophila *lig4* mutants, which are completely defective in C-NHEJ, also survive IR doses in excess of 4000 rads [17]. However, *spn-A* mutants, which lack the Rad51 recombinase required for strand invasion during homologous recombination initiation [42], are highly sensitive to IR [17]. Thus, in Drosophila, HR is the dominant mechanism used to repair IR-induced DSBs.

To test whether pol θ acts to repair IR damage in the absence of HR, we created *mus308 spn-A* double mutants and exposed them to doses of 125–1000 rads. Strikingly, doses as low as 125 rads resulted in almost complete killing of *mus308 spn-A* mutants (Figure 2B). In contrast, *lig4 spn-A* double mutants are only slightly more sensitive than *spn-A* single mutants to IR [17]. Thus, in the absence of HR, pol θ participates in a process crucial for repair of damage caused by ionizing radiation.

**Figure 3:**
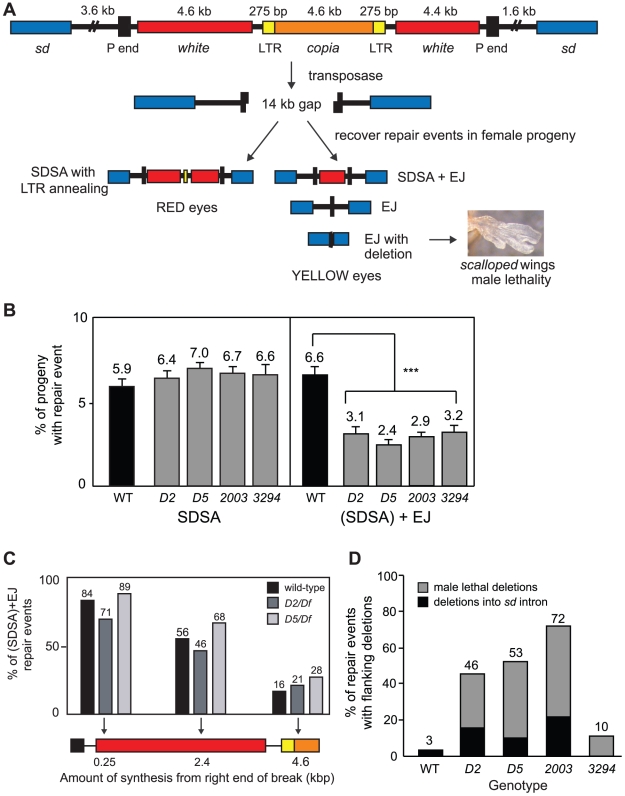
Pol θ is involved in end-joining repair. (A) A *P* element excision assay monitors DSB repair outcomes. The *P(w^a^}* transposon is inserted in a 5.2 kb intron of the essential *scalloped* (*sd*) gene. A copia retrotransposon flanked by long terminal repeats (LTR) is inserted into an exon of the *white* gene, causing reduced expression of *white*. Excision of *P(w^a^}* results in a 14-kilobase gap relative to an intact sister chromatid. Following excision, repair events from the male pre-meiotic germline are recovered in female progeny and the method of repair is inferred from their eye color. SDSA = synthesis-dependent strand annealing, SDSA+EJ = SDSA+end joining, EJ = end joining. Accompanying deletion into *sd* exons (EJ with deletion) results in a scalloped-wing phenotype and/or male lethality. (B) Pol θ mutants are defective specifically in end-joining repair of DSBs. Each bar represents the mean percentage of repair events recovered from independent males possessing the indicated *mus308* allele in trans to *Df(3R)Sz29*. (SDSA)+EJ = repair involving HR initiation followed by end joining or repair involving only end joining. Number of independent males assayed: wild type = 101, *D2* = 155, *D5* = 161, *2003* = 155, *3294* = 100. Error bars represent SEM. (*** indicates p<0.001, Kruskal-Wallis non-parametric ANOVA). p >0.5 for all comparisons between different *mus308* alleles. (C) Synthesis during HR is not impaired in pol θ mutants. Each bar represents the percentage of repair events recovered from yellow-eyed females that showed evidence for repair synthesis of at least the indicated length. Number of independent repair events: wild type = 32, *D2* = 28, *D5* = 53. (D) Pol θ mutants have an increased frequency of DSB repair events with flanking deletions. For all genotypes, small deletions <3.6 kilobases upstream or <1.6 kilobases downstream of *P(w^a^}* were detected by PCR. Large deletions resulted in scalloped-winged female progeny and/or male lethality.

Because interstrand crosslink repair and alternative end joining have been shown to have partially overlapping genetic requirements in mammals [24], [25], we hypothesized that the extreme sensitivity of *mus308 spn-A* mutants to IR might relate to a role of pol θ in an alternative end-joining mechanism. To explore this hypothesis, we tested each *mus308* mutant allele using a site-specific double-strand break repair assay that can distinguish between synthesis-dependent strand annealing (SDSA, a specific type of HR) and end joining (EJ) (Figure 3A) [43]. We have previously shown that the majority of end joining observed in this assay occurs independently of DNA ligase 4, and is therefore a form of alt-EJ [17]. In this system, excision of a *P* element (*P(w^a^}*) located on the *X* chromosome is catalyzed by *P* transposase, resulting in a 14-kilobase gap relative to an undamaged sister chromatid. The DNA ends remaining after excision each have 17-nucleotide non-complementary 3′ single-stranded overhangs [44]. These ends are highly recombinogenic and repair by SDSA is initially favored. However, because repair synthesis in this system is not highly processive, most repair products that are recovered from wild-type flies result from incomplete repair synthesis from one or both sides of the break, followed by end joining of the nascent DNA (SDSA+EJ events) [45]. To quantitate the percentage of repair events that derive from each mechanism, repair events are recovered from male pre-meiotic germline cells by mating individual males to females homozygous for the *P(w^a^}* element. Each of the resulting female progeny represents a single repair event that can be classified by eye color. Red eyed-females inherit a repair event involving homology-dependent synthesis that generated complementary single-stranded regions that subsequently anneal (repair by SDSA). Yellow-eyed females inherit a chromosome that was repaired by EJ or SDSA+EJ mechanisms (these repair events are hereafter referred to as (SDSA)+EJ; for further details, see Materials and Methods).

Overall, the results from the *P(w^a^}* assay indicated that *mus308* mutants are defective in end-joining repair of DSBs. We observed no decrease in the percentage of red-eyed progeny recovered from *mus308* mutant males (Figure 3B), suggesting that SDSA repair is not impaired when pol θ is missing or defective. In contrast, all four *mus308* mutant alleles resulted in a significantly decreased percentage of yellow-eyed progeny relative to wild type (p<0.001, Kruskal-Wallis test). Because yellow-eyed progeny can only result from a repair mechanism involving end joining, these data suggest that pol θ is involved in an end-joining process.

To further demonstrate that pol θ is not involved in DNA synthesis during SDSA, we recovered independent (SDSA)+EJ events in males, isolated genomic DNA, and used PCR to estimate the approximate amount of DNA repair synthesis that occurred prior to end joining. The amount of repair synthesis in (SDSA)+EJ repair products did not differ significantly between wild-type and *mus308* mutant flies (Figure 3C). We conclude that pol θ is not required for DNA synthesis during SDSA, but plays an important role in end-joining repair following aborted SDSA.

### DSB repair in *mus308* mutants frequently results in Rad51-independent genomic deletions

Mutations that abolish end joining in flies cause an increased frequency of genomic deletions during repair of site-specific DSBs [46], [47]. To determine whether mutation of *mus308* also results in repair-associated deletions, we took advantage of the fact that deletions can be easily scored in the *P* element excision assay. Because *P(w^a^}* is inserted in the essential *scalloped* (*sd*) gene, repair events that delete into *sd* exons cause a scalloped-wing phenotype when recovered in heterozygous females and lethality in hemizygous males [48], [49]. We observed a substantial increase in the percentage of deletion-associated repair events isolated from *mus308* mutant males relative to wild type (Figure 3D). Overall, the total percentage of end-joining repair events involving deletions recovered from *mus308* mutants was elevated from 3- to 26-fold over wild type, depending on the *mus308* allele tested.

Previously, we observed a similar deletion-prone phenotype in flies lacking the DmBlm protein, which is involved in repair of DSBs by SDSA [48]. Because our data did not support a role for pol θ in homologous recombination, we expected the deletion-prone phenotype of *mus308* mutants to persist even in SDSA-deficient flies. To confirm this, we assayed repair following *P(w^a^}* excision in *mus308* mutants lacking the Rad51 protein, which renders them unable to carry out HR repair [42], [45].

**Figure 4:**
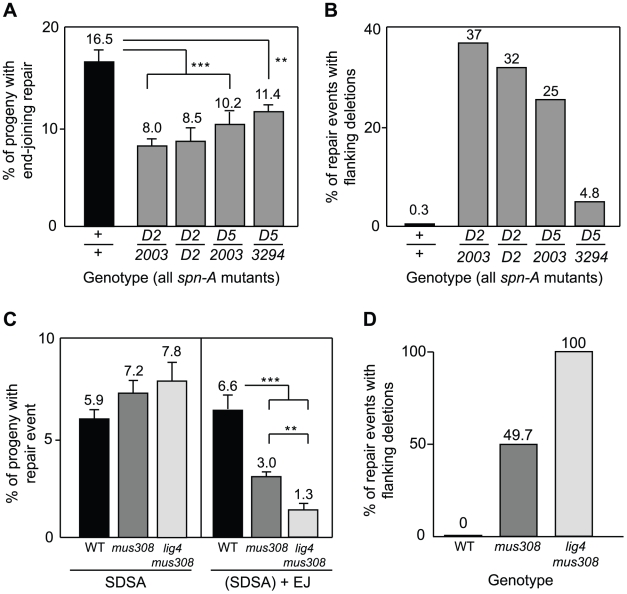
Pol θ–dependent end joining acts independently of HR and C-NHEJ. (A) Decreased end joining in *mus308* mutants does not depend on homologous recombination. Each bar represents the mean percentage of end-joining events recovered from independent males of each genotype. Number of independent males assayed: *spn-A* = 63; *spn-A^057^mus308^D2^/spn-A^057^mus308^2003^* = 216; *spn-A^057^mus308^D2^/spn-A^057^mus308^D2^* = 33; *spn-A^093^mus308^D5^/spn-A^057^mus308^2003^* = 80; *spn-A^093^mus308^D5^/spn-A^093^mus308^3294^* = 128. Error bars represent SEM. ** indicates p<0.01, *** indicates P<0.001 (Kruskal-Wallis non-parametric ANOVA). (B) Genomic deletions during DSB repair in *mus308* mutants do not depend on initiation of HR. Each bar represents the percentage of yellow-eyed female progeny with scalloped wings (representing large flanking deletions) recovered from males of indicated genotype. (C) Mutation of *mus308* in a *lig4^169a^* background further reduces end-joining repair relative to *mus308* single mutants. Each bar represents the mean percentage of repair events recovered from wild type, *mus308^2003^/Df(3R)kar3l*, or *lig4^169a^*; *mus308^2003^*/*Df(3R)kar3l* males. Number of independent males assayed: wild type = 101, *mus308* = 182, *lig4 mus308* = 57. Error bars represent SEM. ** indicates p<0.01, *** indicates P<0.001 (Kruskal-Wallis non-parametric ANOVA). (D) Genomic deletions always occur during DSB repair in *lig4 mus308* mutants. All yellow-eyed repair events recovered from *lig4^169a^*; *mus308^2003^/Df(3R)kar3l* males had large deletions that resulted in male lethality and/or female progeny with scalloped wings. Number of independent repair events assayed: wild type = 101, *mus308* = 167, *lig4 mus308* = 22.

As expected, PCR analysis of repair products showed that SDSA was abolished in both *spn-A* and *spn-A mus308* mutants (data not shown). Approximately 17% of *P(w^a^}* chromosomes recovered from *spn-A* mutant males showed evidence for end joining at the 17-nucleotide overhangs that are created by *P* transposase (Figure 4A and Table 1); the other 83% of *P(w^a^}* chromosomes recovered were presumably uncut. We observed a 30–50% decrease in end-joining repair products in *spn-A mus308* double mutants compared to *spn-A* mutants (p<0.001, Kruskal-Wallis test), confirming a unique role for polθ in end joining when HR is absent. Importantly, mutation of *mus308* still caused an increased percentage of deletions in the absence of Rad51 (Figure 4B). From these data, we conclude that the deletions formed during break repair in *mus308* mutants are not the result of aborted SDSA. Rather, they are a consequence of a deletion-prone repair mechanism that operates in the absence of both SDSA and pol θ-dependent end joining.

**Table 1:** *P(w* a *}* repair junctions recovered from *spn-A^093/057^* mutants.

Type of repair event	Sequence 5′ of break^a^	Microhomology/inserted sequence	Sequence 3′ of break^a^	Number of isolates
**Original sequence**	acccagacCATGATGAAATAACATA	-	TATGTTATTTCATCATGacccagac	-
**Long microhomology** b				
	acccagac	(CATgATGA)^c^	cccagac	14
	acccagac	(CATGA)	cccagac	4
	none^d^	(TGACCCAGAC)	-	2
**Short microhomology**				
	acccagacCATG	(ATG)	TTATTTCATCATGacccagac	1
	acccagacCA	(TGA)	cccagac	1
	acccagacCATGATGAAATAA	(CAT)	Gacccagac	3
	acccagacCATGATGAAATAAC	(AT)	GTTATTTCATCATGacccagac	14
	acccagacCATGATGAAATAACA	(TA)	TGTTATTTCATCATGacccagac	5
	acccagacCATGATGAAA	(TA)	TGTTATTTCATCATGacccagac	1
	acccagacCATGATGAA	(AT)	GTTATTTCATCATGacccagac	1
	none^d^	(T)	TCATGacccagac	1
**Blunt join**				
	acccagacCATGATGAAATA	-	TTATTTCATCATGacccagac	1
**Insertion** e				
	acccagacCATGATGAAATAACATA	A	CATCATGacccagac	1
	acccagacCATGATGA	G	CATCATGacccagac	1
	acccagacCATGATGAAATAACATA	AC	ATGTTATTTCATCATGacccagac	3
	acccagacCATGATGAAATAACAT	GT	TATGTTATTTCATCATGacccagac	1
	acccagacCATGATGA	AC	ATGTTATTTCATCATGacccagac	1
	acccagacCA	AG	ATGacccagac	1
	acccagacCATGATGAAATAACATA	TTC	ATGTTATTTCATCATGacccagac	1
	acccagacCATGATGAAATAACAT	GTTA	TATGTTATTTCATCATGacccagac	1
	acccagacCATGATGAAATAACATA	TTTAT	TGTTATTTCATCATGacccagac	1
	acccagacCATGATGAAATAACAT	TTATCA	TGTTATTTCATCATGacccagac	1
	acccagacCATGATGAAATAACAT	TTAACATAAC	ATGTTATTTCATCATGacccagac	1
	acccagacCATGATGAAATAACATA	TTATTATTATA	TTATTTCATCATGacccagac	1
	acccagacCATGATGAAATAACAT	GAAATAATAAC	ATGTTATTTCATCATGacccagac	1
	acccagacCATGATGAAATAACAT	GTATTACATAAC	ATGTTATTTCATCATGacccagac	1
	acccagacCATGATGAAATAA	TAATAATAATATAA	TATGTTATTTCATCATGacccagac	1
	acccagacCATGATGAAATA	TCATGAAATATCATA	TCATCATGacccagac	1

**a** Uppercase letters represent the 17-nucleotide 3′ single-stranded tails that remain following transposase action, lowercase letters correspond to the 8 base pair target sequence duplicated upon *P* element insertion.

**b** Microhomologies (in parentheses) are sequences that could have been derived from either side of the break site. Long microhomologies were five or more nucleotides, while short microhomologies were three or fewer nucleotides.

**c** represents an 8-nucleotide imperfect microhomology.

**d** indicates a deletion that extends past the 8 base pair target sequence.

**e** Insertions were identified as any sequence not present at the original break site. Templated insertions and corresponding potential templates in flanking sequences are underlined.

**Figure 5:**
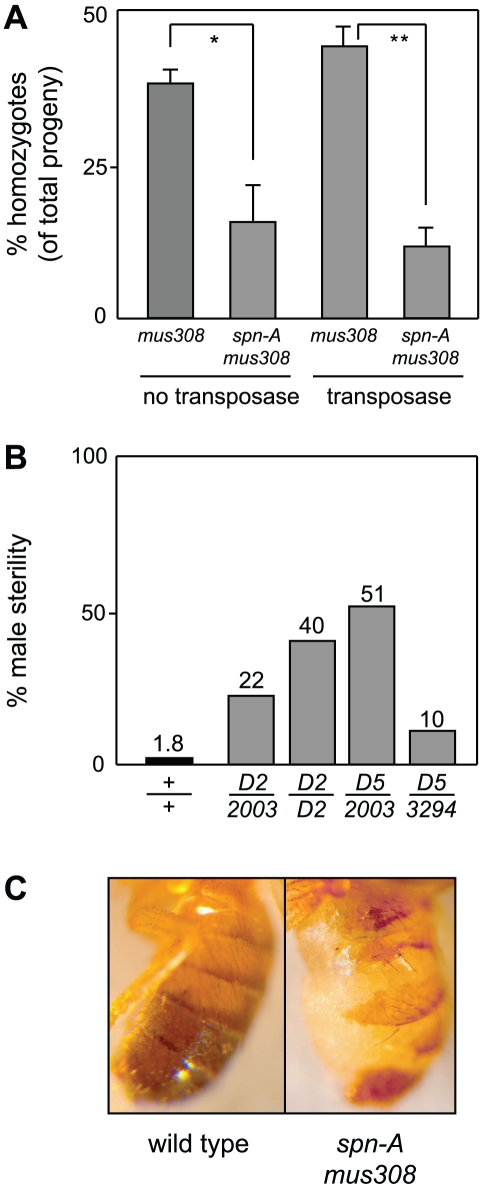
Flies lacking Rad51 and Pol θ have synergistic phenotypic defects. (A) *spn-A mus308* mutants are sub-viable. Females and males with the *P(w^a^}* transposon and heterozygous for the *spn-A^057^* or *spn-A^093^* and *mus308^D2^* or *mus308^D5^* alleles were interbred and the percentage of viable compound heterozygous progeny was determined. The crosses were repeated in the presence of *P* transposase. Each bar represents three independent experiments with at least 300 progeny per experiment. Error bars represent SEM. * indicates p<0.05, ** indicates P<0.01 (unpaired T test). (B) Increased male sterility in *spn-A mus308* double mutant males. Each bar represents the percentage of *spn-A* or *spn-A mus308* males that produced no progeny. Number of independent males assayed: *spn-A* = 57; *spn-A^057^mus308^D2^/spn-A^057^mus308^2003^* = 305; *spn-A^057^mus308^D2^/spn-A^057^mus308^D2^* = 50; *spn-A^093^mus308^D5^/spn-A^057^mus308^2003^* = 102; *spn-A^093^mus308^D5^/spn-A^093^mus308^3294^* = 100. (C) Mutation of both *spn-A* and *mus308* results in external morphological defects such as abdominal cuticle mispatterning (note the severe disruption of normal segmental banding patterns). Pictured are representative wild type and *spn-A^057^mus308^D2^/spn-A^057^mus308^2003^* males in which *P* element excision is occurring.

During the course of these experiments, we made a number of observations suggesting that Rad51 and pol θ act in parallel and distinct DSB repair mechanisms. First, we recovered fewer *spn-A mus308* double mutant males than would be predicted from Mendelian ratios. For example, in crosses between *mus308^D2^* and *mus308^D5^* heterozygotes, 38% of the progeny were *mus308^D2^*/*mus308^D5^* compound heterozygotes. In contrast, only 16% of progeny recovered from parallel matings between *spn-A^057^mus308^D2^* and *spn-A^093^mus308^D5^* mutants were *spn-A mus308* compound heterozygotes (*P*<0.05, Fisher's exact test; Figure 5A). This difference in viability between *mus308* and *spn-A mus308* mutants was even more extreme in flies in which excision of *P(w^a^}* was occurring (*P*<0.01; Figure 5A). In addition, we observed heightened male sterility in various combinations of *spn-A mus308* mutants undergoing *P(w^a^}* transposition, with 51% of the double mutant males unable to produce viable progeny in the most severe allele combination (Figure 5B). Finally, we observed morphological abnormalities, specifically abdominal closure defects and aberrant cuticle banding patterns, in 100% of *spn-A^093^mus308^D5^*/*spn-A^057^mus308^D2^* double mutants (Figure 5C). These defects were more severe in the double mutants experiencing *P(w^a^}* transposition, but were not apparent in either *mus308* or *spn-A* single mutants. From these data, we conclude that Rad51 and pol θ participate in independent pathways required for repair of DSBs that arise during both endogenous developmental processes and during *P* element transposition.

### Pol θ–mediated end joining is distinct from C-NHEJ

*P* element ends are good substrates for DNA ligase 4-independent end joining [17]. Based on the results presented above, it seemed likely that pol θ is involved in an end-joining process different from C-NHEJ. To formally test this, we repeated the *P(w^a^}* assay in *lig4 mus308* double mutants that lack DNA ligase 4 and are unable to repair DSBs by C-NHEJ. Unlike *spn-A mus308* mutants, we observed no viability, fertility, or morphological defects in *lig4 mus308* mutants. We also observed no defect in HR repair in the double mutants (Figure 4C), consistent with results obtained using *mus308* single mutants. In contrast, we observed a further decrease in the percentage of end joining repair products recovered from *lig4 mus308* double mutants relative to *mus308* mutants, from 3.0% to 1.3% (*P*<0.01, Kruskal-Wallis test). Previously, we have shown that end-joining repair of DSBs induced by *P(w^a^}* excision is unaffected in *lig4* mutants [17]. Therefore, the removal of pol θ-mediated end joining reveals a previously hidden role for DNA ligase 4 in the repair of DSBs created by *P* transposase. Strikingly, although only 50% of end-joining products isolated from *mus308* mutants involved large, male-lethal deletions, 100% of end-joining products recovered from *lig4 mus308* mutant males were associated with large deletions (Figure 4D).

From these results, we conclude that at least three distinct mechanisms for end-joining repair exist in Drosophila. One, which corresponds to C-NHEJ, requires DNA ligase 4 and other canonical NHEJ proteins, including XRCC4, Ku70, and Ku80 [46], [47], [50]. Another mechanism, which is at least partially independent of DNA ligase 4, is defined by a requirement for pol θ and corresponds to alt-EJ. Interestingly, alt-EJ appears to be used more frequently than C-NHEJ, at least for the repair of *P* element-induced breaks. In the absence of these two repair processes, a Rad51-independent backup mechanism characterized by extensive genomic deletions operates at low efficiency.

### Pol θ has two distinct functions in alternative end joining

Alt-EJ repair in Drosophila is frequently associated with annealing at microhomologous sequences of more than four nucleotides and with long DNA insertions at repair junctions [8]. To determine whether pol θ-dependent end joining involves either of these types of repair, we sequenced repair junctions obtained from *spn-A* and *spn-A mus308* double mutants following *P(w^a^}* excision. Because we sequenced only one junction per male germline, each junction analyzed represents an independent repair event. Five distinct junction types were identified. Three of these types are characteristic of junctions arising from C-NHEJ in mammalian systems [7]: junctions involving small, 1–3 base pair insertions, junctions involving annealing at 1–3 nucleotide microhomologies, and junctions for which no microhomologies can be identified (apparent blunt end junctions). The other two types of junctions, characteristic of alt-EJ [8], involve annealing at 5–10 nucleotide microhomologous sequences or insertions of more than three base pairs.

**Figure 6:**
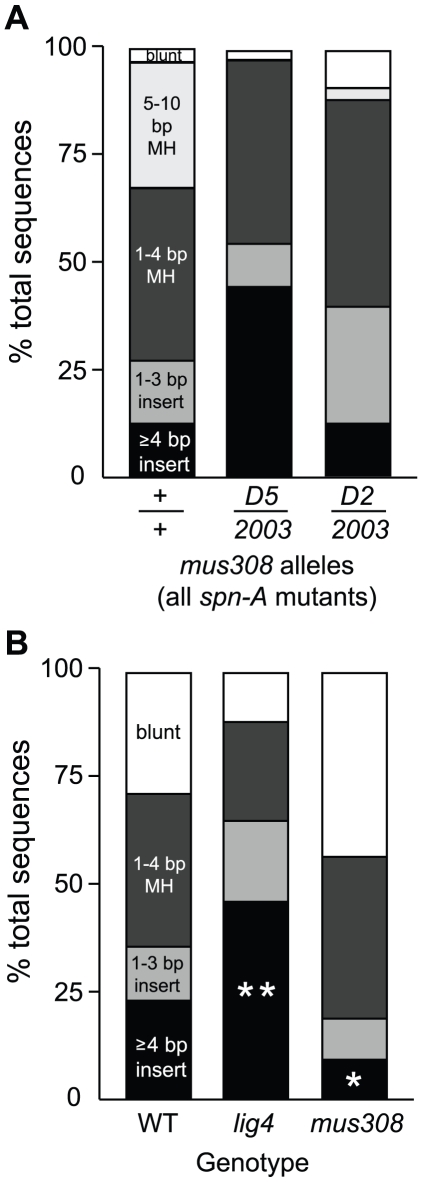
Pol θ is required for two distinct classes of alt–EJ repair products. (A) Annealing at long microhomologies is greatly decreased in pol θ mutants. Repair junctions isolated following *P(w^a^}* excision were amplified from *spn-A* and *spn-A mus308* females of the indicated genotypes and sequences were aligned to the original *P(w^a^}* chromosome. White, blunt joins; hatched, 5–10 bp microhomologies; light gray, 1–4 bp microhomologies; dark gray, 1–3 bp insertions; black, ≥4 bp insertions. Number of junctions sequenced: *spn-A* = 68, *spn-A^093^mus308^D5^*/*spn-A^057^mus308^2003^* = 23, *spn-A^057^mus308^D2^*/*spn-A^057^mus308^2003^* = 25. The percentage of 5–10 bp microhomologies was significantly decreased in both *spn-A mus308* mutants (*P*<0.01, Fisher's exact test). (B) Reduced frequency of ≥4 bp insertions during inaccurate end-joining repair of a chromosomal I-*Sce*I DSB in *mus308* mutants. Complementary ended DSBs were induced by expression of I-*Sce*I endonuclease in the pre-meiotic germline of wild type (n = 70), *lig4^169a^* (n = 83), and *mus308^2003^/Df(3R)kar-Sz29* (n = 57) males, and independent inaccurate end-joining repair junctions were sequenced. White, blunt joins; light gray, 1–4 bp microhomologies; dark gray, 1–3 bp insertions; black, ≥4 bp insertions. No >4 bp microhomologies are available near the DSB in this system. * indicates *P* = 0.03, ** indicates *P*<0.01, relative to wild type (Fisher's exact test).

Approximately 58% of junctions from *spn-A* mutants showed structures considered typical of C-NHEJ repair, while 29% involved annealing at 5–10 nucleotide microhomologies and 13% had insertions of greater than three base pairs (Figure 6A and Table 1). Potential templates for the larger insertions could almost always be identified in flanking sequences. These insertions may be analogous to T-nucleotides that have been observed at translocation breakpoint junctions isolated from certain human cancers [21]–[23].

When we sequenced repair junctions from *spn-A mus308* mutants, we observed two distinct patterns, depending on the *mus308* alleles used. For both the *D2/2003* and *D5/2003* allele combinations, the percentage of junctions involving annealing at long microhomologies was significantly decreased (*P*<0.01, Fisher's exact test; Figure 6A, Table 2, and Table 3). Only 12% of *D2/2003* junctions possessed an insertion greater than three base pairs, compared to 44% of junctions recovered from males with the *D5* and *2003* alleles. In addition, most insertions isolated from *D5/2003* males were highly complex and had multiple copies of imperfect repeats of T-nucleotides. Similar results were obtained with the *D5/3294* allele combination (data not shown). An overall comparison of insert length showed that flies with wild-type *mus308* alleles had an average insert length of 5.5 nucleotides, compared to 3.8 nucleotides for *D2/2003* mutants and 13.3 nucleotides for *D5/2003* mutants.

**Table 2:** *P(w* a *}* repair junctions recovered from *spn-A^093/057^ mus308^D5/2003^* mutants.

Type of repair event	Sequence 5′ of break	Microhomology/inserted sequence	Sequence 3′ of break	Number of isolates
**Original sequence**	acccagacCATGATGAAATAACATA	-	TATGTTATTTCATCATGacccagac	-
**Short microhomology** a				
	acccagacCATGATGAAATAA	(CAT)	CATGacccagac	2
	acccagacCATG	(ATG)	TTATTTCATCATGacccagac	1
	acccagacCATGATGAAATAAC	(AT)	GTTATTTCATCATGacccagac	5
	acccagacCATGATGAAATAACA	(TA)	TGTTATTTCATCATGacccagac	2
**Blunt join**				
	acccagacCATGATGAAATAACATA	-	TTATTTCATCATGacccagac	1
**Insertion** b				
	acccagacCATGATGAAATAACAT	G	TGTTATTTCATCATGacccagac	1
	acccagacCATGATGAAATAACATA	AC	ATGTTATTTCATCATGacccagac	1
	acccagacCATGATGAAATAACAT	GTTA	TATGTTATTTCATCATGacccagac	1
	acccagacCATGATGAAATAACATA	TGTA	TATGTTATTTCATCATGacccagac	1
	acccagacCATGATGAAATAACA	GTGAA	ATGTTATTTCATCATGacccagac	1
	acccagacCATGATGAAATAACAT	GTTATGT	TATGTTATTTCATCATGacccagac	1
	acccagacCATGATGAAATAACAT	GTTATACA	TATGTTATTTCATCATGacccagac	1
	acccagacCATGATGAAATAACATA	TATGTTATAACA	TATGTTATTTCATCATGacccagac	1
	acccagacCATGATGAAATAA	TCATGTTATTTC	ATGTTATTTCATCATGacccagac	1
	acccagacCATGATGAAATAACATA	TAACATGAATAAC	ATGTTATTTCATCATGacccagac	1
	acccagacCATGATGAAATAACATA	TATAATGTTATAACATATAACATATGTTATGAAATAATAACA	TATGTTATTTCATCATGacccagac	1
	acccagacCATGATGAAATAACAT	CATCATTTATCATTTATTATTATTATTATTTATTATTATTTATTATTTA	TTATTTCATCATGacccagac	1

**a** Microhomologies (in parentheses) are sequences that could have been derived from either side of the break site.

**b** Insertions were identified as any sequence not present at the original break site. Templated insertions and corresponding potential templates in flanking sequences are underlined.

**Table 3:** *P(w* a *}* sequences recovered from *spn-A^093/057^ mus308^D2/2003^* mutants.

Type of repair event	Sequence to left of break	Microhomology/inserted sequence	Sequence to right of break	Number of isolates
**Original sequence**	acccagacCATGATGAAATAACATA	-	TATGTTATTTCATCATGacccagac	-
**Long microhomology** a				
	acccagac	(CATgATGA)	cccagac	1
**Short microhomology**				
	acccagacCATGATGAAATAAC	(AT)	GTTATTTCATCATGacccagac	7
	acccagacCATGATGAAATAACA	(TA)	TGTTATTTCATCATGacccagac	3
	acccagacCATGATGA	(AT)	GTTATTTCATCATGacccagac	1
	acccagacCATGATGAAATAACA	(T)	TATTTCATCATGacccagac	1
**Blunt join**				
	acccagacCATGATGAAATAACATA	-	TATGTTATTTCATCATGacccagac	1
	acccagacCATGATGAAATAACAT	-	TATGTTATTTCATCATGacccagac	1
**Insertion** b				
	acccagacCATGATGAAATAACAT	T	TATGTTATTTCATCATGacccagac	1
	acccagacCATGATGAAATAACATA	CA	ATGTTATTTCATCATGacccagac	1
	acccagacCATGATGAAATAACATA	AC	ATGTTATTTCATCATGacccagac	1
	acccagacCATGATGAAATAACATA	TA	TATGTTATTTCATCATGacccagac	1
	acccagacCATGATGAAATAA	TGT	TATGTTATTTCATCATGacccagac	1
	acccagacCATGATGAAATAACATA	TGT	TATGTTATTTCATCATGacccagac	2
	acccagacCATGATGAAATAACATA	ACATAA	ATGTTATTTCATCATGacccagac	1
	acccagacCATGATGAAATAACATA	TATACCG	TATGTTATTTCATCATGacccagac	1
	acccagacCATGATGAAATAACATA	TGTTATAAC	ATGTTATTTCATCATGacccagac	1

**a** Microhomologies (in parentheses) are sequences that could have been derived from either side of the break site.

**b** Insertions were identified as any sequence not present at the original break site. Templated insertions and corresponding potential templates in flanking sequences are underlined.

In summary, both *mus308* mutant combinations significantly abrogated annealing at long microhomologies during alt-EJ repair. However, we observed a distinct difference in repair junctions recovered from males harboring the *D2* allele, which greatly reduces overall pol θ protein levels [38], compared to flies with the *D5* allele, which alters a conserved residue near the helicase-like domain. These results suggest that pol θ has two distinct functions in alt-EJ: one that promotes the annealing of long microhomologous sequences during end alignment, and another that is responsible for complex T-nucleotide insertions. Flies with the *D2* allele are impaired in their ability to carry out both the annealing and insertion functions, whereas flies possessing the *D5* separation-of-function allele cannot perform the microhomology annealing function but can still produce complex insertions.

### Pol θ operates in alternative end joining of complementary-ended double-strand breaks

*P* element-induced breaks are unique in that they possess 17-nucleotide non-complementary ends that are poor substrates for C-NHEJ. To test whether the results obtained with *P* elements can be generalized to other types of breaks, we used the I-*Sce*I endonuclease and the previously characterized *[Iw]7* reporter construct [50] to create site-specific DSBs in wild-type flies and flies lacking either DNA ligase 4 or pol θ. I-*Sce*I produces a DSB with 4-nucleotide complementary overhangs that can be directly ligated through a C-NHEJ mechanism [50], [51]. Accurate repair regenerates the original I-*Sce*I recognition sequence, which can then be cut again, while inaccurate end-joining repair abolishes further cutting. We utilized an *hsp70* or ubiquitin-driven I-*Sce*I construct integrated on chromosome *2* to drive high levels of I-*Sce*I expression [50], [52]. Nearly 100% of repair events that we recovered involved gene conversion (HR repair from the homologous chromosome) or inaccurate end-joining (data not shown). In the *[Iw]7* system, both gene conversion events and large deletions that remove the *white* marker gene are phenotypically indistinguishable. PCR analysis confirmed that many repair events recovered from *mus308* mutants involved large deletions (>700 base pairs, data not shown). Our subsequent analysis focused on the characterization of repair events involving smaller deletions.

Twenty-three percent of I-*Sce*I repair junctions isolated from wild-type flies possessed insertions of more than 3 base pairs (Figure 6B). This percentage was significantly increased to 46% in *lig4* mutants (*P*<0.01, Fisher's exact test), consistent with increased use of alt-EJ in the absence of C-NHEJ. If pol θ plays a general role in insertional mutagenesis during alt-EJ repair, one would predict that the frequency and length of insertions following I-*Sce*I cutting should decrease in *mus308* mutants. Indeed, the percentage of large insertions decreased to 9% in *mus308* mutants (*P* = 0.03, Fisher's exact test). Wild-type flies had an average insertion length of 7.6 base pairs, compared to 4.2 base pairs for *mus308* mutants. Strikingly, no *mus308* insertion was longer than twelve base pairs, while insertions of more than twenty base pairs occurred in both wild type and *lig4* mutants. Because microhomologies of greater than four base pairs are not present near the I-*Sce*I cut site in this construct, repair involving annealing at long microhomologies was not observed.

Surprisingly, the total percentage of repair junctions with short, 1–3 base pair insertions was not decreased in *lig4* mutants relative to wild type (17% vs. 13%, respectively). Furthermore, the percentage of junctions involving annealing at 1–3 nucleotide microhomologies was also similar between the two genotypes (25% for *lig4* mutants vs. 34% for wild type). These two types of junctions have historically been associated with ligase 4-dependent C-NHEJ repair. Our results suggest that this may not be the case. Indeed, a fine-level sequence analysis of I-*Sce*I repair junctions that we have recently conducted suggests that alt-EJ may produce C-NHEJ-like junctions in certain sequence contexts [53]. Nevertheless, our data obtained using two independent site-specific DSB repair assays strongly suggest that C-NHEJ and alt-EJ represent at least partially independent mechanisms for the repair of DSBs and that pol θ plays an important role in the generation of T-nucleotide insertions during alt-EJ repair of both *P* element and I-*Sce*I-induced breaks.

## Discussion

Several studies have identified proteins important for end-joining repair of DSBs in the absence of C-NHEJ factors in yeasts [11], [54], [55]. More recently, the Mre11 protein has been identified as an important alt-EJ component in vertebrate systems [10], [56]–[59]. However, much remains uncertain about the genetics or mechanisms of alt-EJ in metazoans. Our previous work demonstrated that end-joining repair of *P* element-induced breaks can occur independently of DNA ligase 4, suggesting the presence of a highly active alternative end-joining mechanism in Drosophila [17]. We have now identified the *mus308* gene, encoding DNA polymerase theta, as a critical component of alt-EJ. Pol θ-dependent alt-EJ operates in parallel to C-NHEJ to promote repair of both *P* element and I-*Sce*I-induced breaks. Because we observed similar alt-EJ defects with four different *mus308* mutant alleles, several of which were studied *in trans* to at least two independently-derived deficiencies, we consider it highly unlikely that the phenotypes we observed are due to second-site mutations or other differences in genetic background.

Importantly, pol θ does not appear to participate in homology-directed repair. HR repair of DSBs following *P(w^a^}* excision is thought to proceed largely through synthesis-dependent strand annealing (SDSA) [60]. We observed that SDSA frequencies in the *P(w^a^}* assay were similar in wild type and *mus308* mutants. Although we did not formally test for a role of pol θ in single-strand annealing (SSA), a non-conservative HR pathway that involves annealing at direct repeats larger than 25 nucleotides, a separate study demonstrated that pol θ has no effect on SSA repair [47]. In addition, comparison of the DNA sequences located 3 kilobases to either side of *P(w^a^}* by BLAST does not reveal any significant similarities of more than 20 nucleotides. Therefore, it seems unlikely that the repair observed in *mus308* mutants arose through an SSA mechanism.

Three findings suggest that *mus308*-dependent alt-EJ is an important repair option for both cell and organism survival in flies, particularly in the absence of homologous recombination. First, *spn-A mus308* double mutants are sub-viable and have severe defects in adult abdominal cuticle closure, consistent with a high level of apoptosis in rapidly proliferating histoblasts during pupariation. Second, *spn-A mus308* double mutant males undergoing *P(w^a^}* excision have up to 30-fold increased sterility relative to *spn-A* mutants. Third, *mus308* mutant males undergoing I-*Sce*I cutting show premature sterility and produce few progeny. The few germline repair events that are recovered from each male are frequently clonal, suggesting extensive germline apoptosis (A. Yu, unpublished data).

### Evidence for two functions of pol θ in alternative end joining

Pol θ orthologs characterized from a variety of metazoans possess both helicase-like and DNA polymerase domains [27]–[31], [35]. Pol θ purified from both Drosophila and human cells has a Pol I-like polymerase activity and single-stranded DNA-dependent ATPase activity [30], [38]. However, DNA helicase activity of the purified protein remains to be demonstrated. Although our experiments did not formally test for helicase activity of pol θ, our results are consistent with pol θ having a DNA unwinding or strand displacement function. Flies with the *D5* and *3294* mutations (located in or near the conserved helicase domain) produce repair products with complex T-nucleotide insertions but not products involving annealing at long microhomologies. The *D5* and *3294* alleles may therefore encode proteins that retain polymerase activity but lack unwinding activity, resulting in an inability to expose internal microhomologous sequences. Because the microhomologies used in repair following *P* element excision are often located in the 17-nucleotide 3′ single-stranded tails, pol θ may also be important for the unwinding of secondary structures that form in single-stranded DNA. Alternatively, the DNA-dependent ATPase activity demonstrated by pol θ might represent an annealing function of the protein that is required during alt-EJ. Such an annealing activity was recently described for the human HARP protein, which is able to displace stably bound replication protein A and rewind single-stranded DNA bubbles [61].

One notable aspect of alt-EJ in Drosophila is the large percentage of repair junctions with templated insertions. These insertions may be “synthesis footprints” that are formed during the cell's attempt to create microhomologous sequences that can be used during the annealing stage of alt-EJ when suitable endogenous microhomologies are not present or are not long enough to allow for stable end alignment. Indeed, analysis of the insertions from I-*Sce*I repair junctions suggests a model involving local unwinding of double-stranded DNA and iterative synthesis of 3–8 nucleotide runs [53]. The *P(w^a^}* repair junctions isolated from *mus308^D2^/mus308^2003^* mutants are consistent with an important (but not exclusive) role for the polymerase domain of pol θ in the synthesis of T-nucleotides.

**Figure 7:**
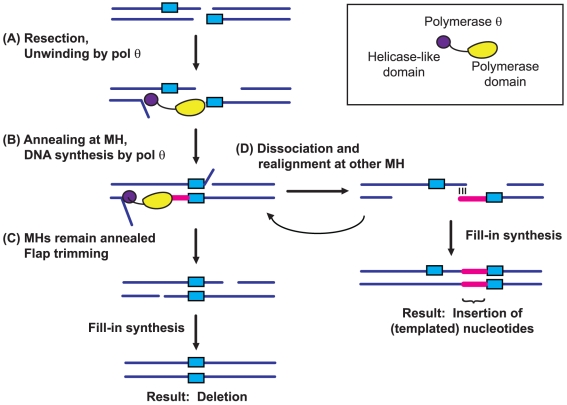
Model for pol θ function in alt–EJ. Pol θ is drawn as a bipartite protein, with a helicase domain and a polymerase domain separated by a flexible linker region. (A) Initially, the helicase activity of pol θ unwinds short stretches of double-stranded DNA to expose pre-existing microhomologous sequences. (B) These microhomologies (MH) are used to align the broken ends to provide a template for pol θ polymerase activity. The unwinding activity could also serve to make the polymerase more processive. (C) Processing of the ends and ligation results in repair accompanied by a deletion. (D) In cases where the ends do not remain stably aligned, annealing at other microhomologies closer to the break site could result in the insertion of T-nucleotides. Multiple rounds of unwinding, synthesis, and alignment could result in the complex insertions that are often observed in alt-EJ in flies.

We speculate that pol θ may be involved in both DNA unwinding and repair synthesis during alt-EJ (Figure 7). Linking these two activities in one protein would provide a convenient mechanism for creating longer microhomologies that could increase the thermodynamic stability of aligned ends prior to the action of a DNA ligase. Studies based on the crystal structure of a dual function NHEJ polymerase-ligase protein found in *Mycobacterium tuberculosis* suggest that a synaptic function for an NHEJ polymerase is plausible [62]. Because ligase 4 is not involved in alt-EJ in Drosophila, another ligase must be involved in the ligation step. Studies from mammalian systems have identified DNA ligase 3 as a likely candidate [63], [64].

### Potential roles of pol θ in DNA interstrand crosslink repair

Pol θ was originally identified in Drosophila based on the inability of *mus308* mutants to survive exposure to chemicals that induce DNA interstrand crosslinks. A crucial question posed by our findings is whether pol θ performs a common function during the repair of both DSBs and interstrand crosslinks. The *C. elegans* pol θ ortholog, POLQ-1, is also required for resistance to interstrand crosslinks and acts in a pathway that is distinct from HR but depends on *CeBRCA1* [28]. In *S. cerevisiae*, several repair mechanisms are utilized during interstrand crosslink repair, including nucleotide excision repair (NER), HR, and translesion synthesis [65], [66]. Given our results and the findings from *C. elegans*, it seems unlikely that the role of pol θ in interstrand crosslink repair involves a function in HR.

In human cells, exposure to agents that induce interstrand crosslinks causes a shift in repair mechanisms that leads to increased use of non-conservative pathways associated with complex insertions and deletions [67]. Furthermore, interstrand crosslinks can cause frequent recombination between direct repeats [68], [69], suggesting that single-strand annealing may provide a viable mechanism for interstrand crosslink repair. The single-strand annealing model of interstrand crosslink repair posits that NER-independent recognition and processing of the crosslinked DNA is followed by generation of single-stranded regions flanking the crosslink and annealing at repeated sequences. Because alt-EJ frequently proceeds through annealing at short direct repeats, it is tempting to speculate that the role of pol θ in interstrand crosslink repair might be to expose and/or promote the annealing of microhomologous single-stranded regions that flank the crosslinked DNA. Consistent with this model, the initial incision step made after recognition of the interstrand crosslink remains normal in *mus308* mutants [26]. Alternatively, pol θ might utilize its polymerase activity and nearby flanking sequences as a template to synthesize short stretches of DNA that could be used to span a single-stranded gap opposite of a partially excised crosslink. Such a model has been proposed to explain the formation of microindels in human cancers [70]. We are currently testing these two models using helicase- and polymerase-specific *mus308* mutant alleles.

### Pol θ and alternative end joining: promoting genome (in)stability

Although it seems counterintuitive, alt-EJ likely functions in some situations to promote genome stability. As evidence of this, we found that DSB repair following *P* element excision in *mus308* mutant flies frequently results in genomic deletions of multiple kilobases. A similar deletion-prone phenotype was previously observed in *mus309* mutants, which lack the Drosophila BLM ortholog [43], [71]. Epistasis analysis demonstrated that the *mus309* deletion phenotype depends on Rad51, implying that DmBlm acts after strand invasion during HR and that the deletions observed in *mus309* mutants are likely a result of failed SDSA [48]. In contrast, the deletions observed in *mus308* mutants do not depend on Rad51, demonstrating that the function of pol θ in DSB repair is independent of HR. The deletion phenotype is exacerbated in *lig4 mus308* double mutants, suggesting that C-NHEJ and alt-EJ represent two parallel mechanisms to prevent deletions. In the absence of these two end-joining options, resection at the broken ends may continue unchecked, resulting in extensive genomic deletions that are generated by an unknown Rad51-independent repair mechanism. Therefore, both C-NHEJ and alt-EJ function to prevent overprocessing of broken DNA ends and extreme degradation of the genome. Microhomology-mediated end joining, which shares many features with alt-EJ, has been proposed to perform a similar function in urothelial cells [72].

Nonetheless, alternative end-joining repair can also be genome destabilizing, as demonstrated by an increasing number of reports linking it to cancer. We have shown that complex insertions observed in alternative end-joining products are more frequent in flies possessing pol θ. These insertions, which are often combinations of nucleotides derived from several templates inserted in both direct and reverse-complement orientations, are remarkably similar to T-nucleotide insertions found in translocation breakpoints reconstructed from follicular and mantle cell lymphomas (reviewed in [73]). Therefore, if pol θ also functions in alternative end joining and T-nucleotide generation in mammals, it might be an important factor involved in translocation formation.

A recent study suggests that pol θ levels are tightly regulated in humans and that loss of this regulation may promote cancer progression [37]. The protein is primarily found in lymphoid tissues but is upregulated in lung, stomach, and colon cancers. Furthermore, high levels of pol θ expression correlate with poorer clinical outcomes. Intriguingly, pol θ is regulated by endogenous siRNAs in Drosophila [74], [75], although the significance of this regulation is currently unclear. We suggest that polθ-mediated alt-EJ serves as a medium-fidelity repair option used by cells when precise repair cannot be carried out for any number of reasons. As such, it prevents extreme loss of genetic information. However, its error-prone nature requires tight regulation, which, when lost, may lead to excessive inaccurate repair and ultimately, carcinogenesis.

### Conclusions

The results described here establish that Drosophila pol θ plays two distinct roles in an alternative end-joining mechanism operating in parallel to canonical DNA ligase 4-mediated C-NHEJ. This novel finding lays the groundwork for future studies focusing on the specific roles of the pol θ helicase-like and polymerase domains in alt-EJ and DNA interstrand crosslink repair. Whether pol θ plays a similar role in alt-EJ in other organisms, including mammals, remains to be determined. Regardless, these studies reveal an unexpected role for DNA polymerase θ that is required for genomic integrity in Drosophila and possibly other metazoans.

## Materials and Methods

### Drosophila stocks and *mus308* alleles

All flies were maintained on standard cornmeal-based agar food and reared at 25°C. The *mus308 D2* and *D5* stocks were obtained from the Bloomington Stock Center and the *2003* and *3294* stocks were from the Zuker collection [76]. To identify mutations in these stocks, genomic DNA was isolated from flies harboring each allele in trans to *Df(3R)Exel6166* and PCR was performed with primers specific to overlapping regions of the entire coding sequence. PCR products were sequenced and the sequence was compared to the Drosophila reference sequence release 5.10. Sequence changes unique to each allele were verified by sequencing in both orientations. The *lig4^169a^* [17], *spn-A^093^* and *spn-A^057^* [42] stocks harbor null alleles of DNA ligase 4 and Rad51, respectively.

### Mutagen sensitivity studies

For mechlorethamine sensitivity assays, balanced, heterozygous parents were crossed to *Df(3R)Exel6166* and allowed to lay eggs in vials containing 10mL of food for three days, after which they were moved to new vials for two additional days. The first vials were treated with 250µL of 0.005% mechlorethamine dissolved in ddH_2_O, while the second vials were treated only with ddH_2_O. Survival was calculated as the number of homozygous mutant adults divided by the total number of adults that eclosed within 10 days of treatment. Ratios were normalized to untreated controls for each set of vials (five to eight sets of vials were counted for each experiment). For ionizing radiation sensitivity assays, heterozygous parents laid eggs on grape-juice agar plates for 12 hr. Embryos developed at 25°C until larvae reached third-instar stage, at which point they were irradiated in a Gammator 1000 irradiator at a dose rate of 800 rads/min and larvae were transferred to food-containing bottles. Relative survival rates were calculated as above.

### P(w^a^} assay

Repair of DNA double-strand breaks was monitored after excision of the *P(w^a^}* transposon as described previously [43], [77]. *P(w^a^}* was excised in males using a second chromosome transposase source (*CyO, H(w^+^,Δ2–3}*) and individual repair events were recovered in female progeny over an intact copy of *P(w^a^}*. Females with two copies of *P(w^a^}* have apricot eyes [78]. Progeny with red eyes possess a repair event involving HR with annealing of the *copia* LTRs. A fraction of apricot-eyed females also possess HR repair events, but these cannot be distinguished from chromosomes in which no excision event occurred (using the *CyO, H(w^+^,Δ2–3}* transposase source, ∼80% of apricot-eyed female progeny inherit a non-excised *P(w^a^}* element). Yellow-eyed females harbor a repair event in which repair is completed by end joining.

For each genotype, at least 50 individual male crosses were scored for eye color of female progeny lacking transposase. The percentage of progeny from each repair class was calculated on a per vial basis, with each vial representing a separate experiment. Statistical comparisons were done with a Kruskal-Wallis non-parametric ANOVA followed by Dunn's multiple comparisons test using InStat3 (GraphPad).

For analysis of HR synthesis tract lengths, genomic DNA was purified and PCR reactions were performed as in [43], using primer pairs with the internal primer located 250, 2420, and 4674 base pairs from the cut site at the 5′ end of *P(w^a^}*.

For deletion analysis, the percentage of females with scalloped wings was calculated relative to all yellow-eyed females counted. The percentage of male lethal and small (0.1–3.6 kb) deletions was calculated based on a subset of yellow-eyed females (one from each original male parent) that were individually crossed to males bearing the *FM7w* balancer. Vials for which no white-eyed male progeny were recovered were scored as male lethal. Some of the male lethal events also caused a scalloped-wing phenotype in heterozygous females. For those that did not, testing to ensure that the male lethality was due to deletion of *scalloped* coding sequence was performed by recovering the repaired chromosomes *in trans* to the hypomorphic *sd^1^* mutation [79] and scoring for a scalloped-wing phenotype. Repair events which could be recovered in males were subjected to PCR analysis, using primers internal to *P(w^a^}* [43], to detect small deletions into one or both introns of *sd*.

### I-SceI break repair assay

Repair of I-*Sce*I mediated DNA double strand breaks was studied in the context of the chromosomally integrated *[Iw]7* construct [52], which contains a single target site for the I-*Sce*I endonuclease. DSBs were induced in the male pre-meiotic germline by crossing females harboring *[Iw]7* to males expressing the I-*Sce*I endonuclease from a second chromosomal location under the control of either the hsp70 promoter (70[I-*Sce*I]1A) [52] or the ubiquitin promoter (UIE[I-*Sce*I]2R) [50]. Independent inaccurate end-joining repair events from the male pre-meiotic germline were recovered in male progeny and DNA was isolated for analysis [80]. PCR was performed using primers PE5′ (GATAGCCGAAGCTTACCGAAGT) and jn3′b (GGACATTGACGCTATCGACCTA) to amplify a 1.3 kb fragment of the *[Iw]7* construct including the I-*Sce*I target site. Products were gel purified (GenScript) and sequencing of PCR products was performed using the PE5′ primer. Sequences were aligned using ClustalW or by manual inspection against sequence obtained from an uncut *[Iw]7* construct. Statistical comparisons were done using Excel and SPSS.

## Supporting Information

Figure S1 Polymerase theta orthologues from various metazoans. The conserved helicase-like (blue oval) and polymerase (pink box) domains are indicated. All of the orthologs have additional conserved regions in the N and C-termini (white boxes), separated by a variable-length linker region.
(0.51 MB PPT)

Figure S2 Sequence changes in different *mus308* mutant alleles, compared to the Drosophila reference genome sequence. Allele-specific changes are highlighted in yellow. ^1^ +1 corresponds to the ‘A’ in the start codon of *mus308*.
(0.11 MB PPT)

Figure S3 Alignment of conserved N-terminus of *mus308* orthologs. Dmel, *Drosophila melanogaster*; Agam, *Anopheles gambia*; Mmus, *Mus musculus*; Hsap, *Homo sapiens*; Drer, *Danio rerio*; Atha, *Arabidopsis thaliana*; Cele, *Caenorhabditis elegans*. Conserved amino acids are indicated below each alignment. The red arrow corresponds to the G621S substitution in the *3294* allele, the blue arrow corresponds to the P781L substitution in the *D5* allele.
(0.19 MB PPT)
