# Supplementary material for: Dual Roles for DNA Polymerase Theta in Alternative End-Joining Repair of Double-Strand Breaks in Drosophila
Source: PLoS Genet. 2010 Jul 1;6(7):e1001005. doi: 10.1371/journal.pgen.1001005 (PMC2895639; doi:10.1371/journal.pgen.1001005)
Supplement: Figure S1 — Polymerase theta orthologues from various metazoans. The conserved helicase-like (blue oval) and polymerase (pink box) domains are indicated. All of the orthologs have additional conserved regions in the N and C-termini (white boxes), separated by a variable-length linker region. (0.51 MB PPT) [file pgen.1001005.s001.ppt]

## Slide 1
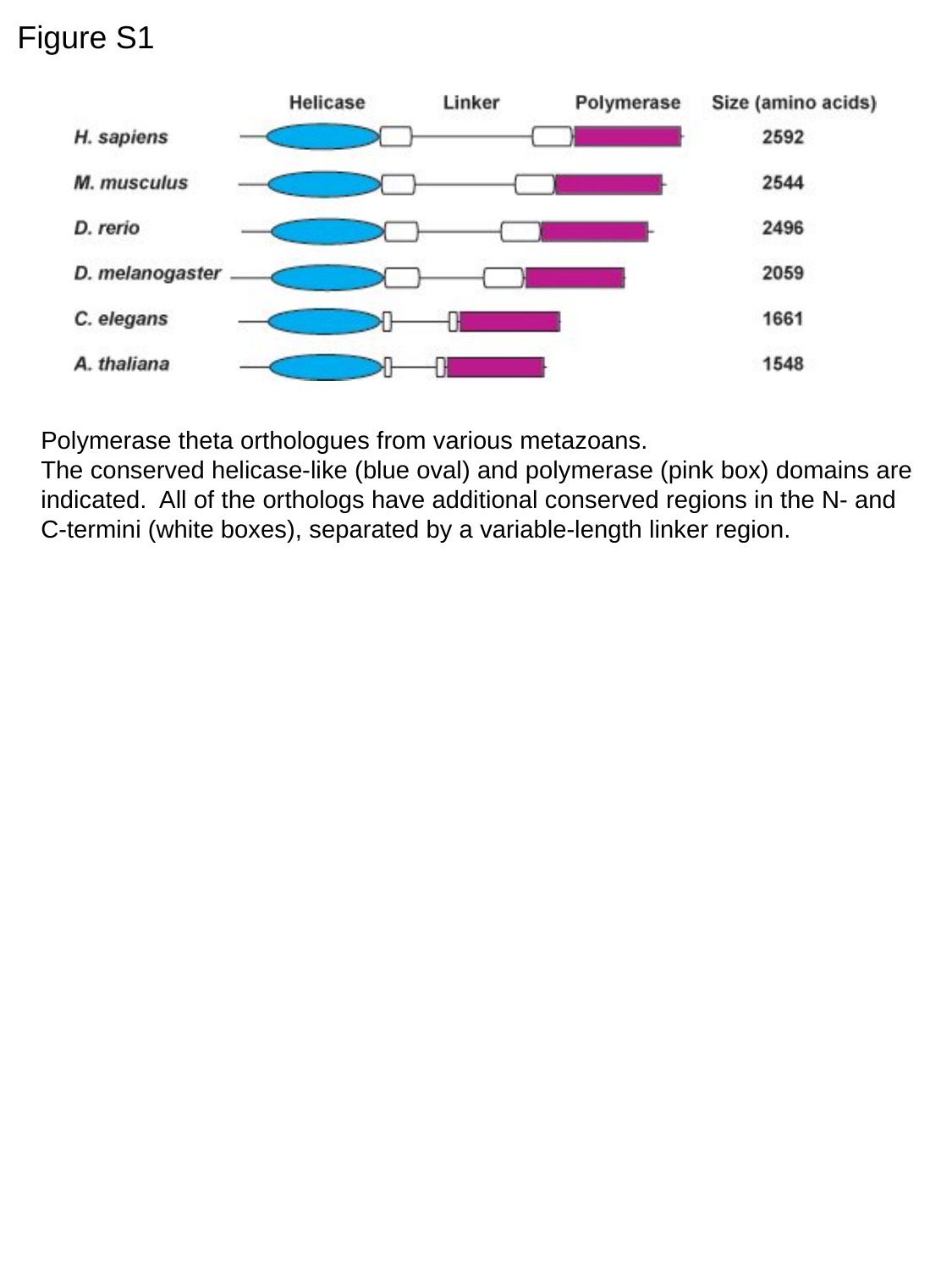

Figure S1
Polymerase theta orthologues from various metazoans.
The conserved helicase-like (blue oval) and polymerase (pink box) domains are
indicated. All of the orthologs have additional conserved regions in the N- and
C-termini (white boxes), separated by a variable-length linker region.
