# Supplementary material for: Dual Roles for DNA Polymerase Theta in Alternative End-Joining Repair of Double-Strand Breaks in Drosophila
Source: PLoS Genet. 2010 Jul 1;6(7):e1001005. doi: 10.1371/journal.pgen.1001005 (PMC2895639; doi:10.1371/journal.pgen.1001005)
Supplement: Figure S2 — Sequence changes in different mus308 mutant alleles, compared to the Drosophila reference genome sequence. Allele-specific changes are highlighted in yellow. 1 +1 corresponds to the ‘A’ in the start codon of mus308. (0.11 MB PPT) [file pgen.1001005.s002.ppt]

## Slide 1
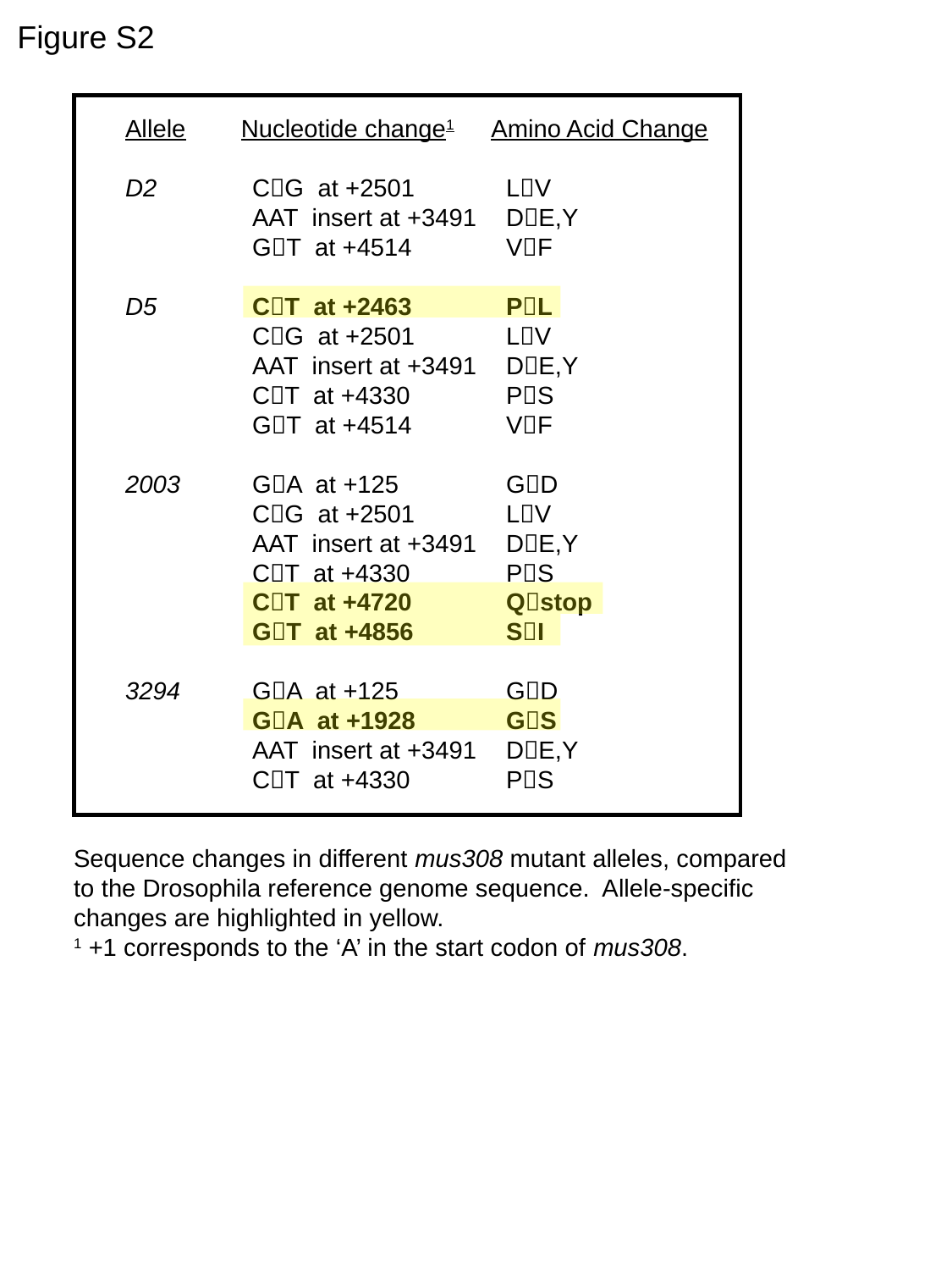

Figure S2
Allele
D2	CG at +2501	LV
	AAT insert at +3491	DE,Y
	GT at +4514	VF
D5	CT at +2463	PL
	CG at +2501	LV
 	AAT insert at +3491	DE,Y
	CT at +4330	PS
	GT at +4514	VF
2003	GA at +125	GD
	CG at +2501	LV
	AAT insert at +3491	DE,Y
	CT at +4330	PS
	CT at +4720	Qstop
	GT at +4856	SI
3294	GA at +125	GD
	GA at +1928	GS
	AAT insert at +3491	DE,Y
	CT at +4330	PS
 Nucleotide change1
 Amino Acid Change
Sequence changes in different mus308 mutant alleles, compared
to the Drosophila reference genome sequence. Allele-specific
changes are highlighted in yellow.
1 +1 corresponds to the ‘A’ in the start codon of mus308.
