# Supplementary material for: Dual Roles for DNA Polymerase Theta in Alternative End-Joining Repair of Double-Strand Breaks in Drosophila
Source: PLoS Genet. 2010 Jul 1;6(7):e1001005. doi: 10.1371/journal.pgen.1001005 (PMC2895639; doi:10.1371/journal.pgen.1001005)
Supplement: Figure S3 — Alignment of conserved N-terminus of mus308 orthologs. Dmel, Drosophila melanogaster; Agam, Anopheles gambia; Mmus, Mus musculus; Hsap, Homo sapiens; Drer, Danio rerio; Atha, Arabidopsis thaliana; Cele, Caenorhabditis elegans. Conserved amino acids are indicated below each alignment. The red arrow corresponds to the G621S substitution in the 3294 allele, the blue arrow corresponds to the P781L substitution in the D5 allele. (0.19 MB PPT) [file pgen.1001005.s003.ppt]

## Slide 1
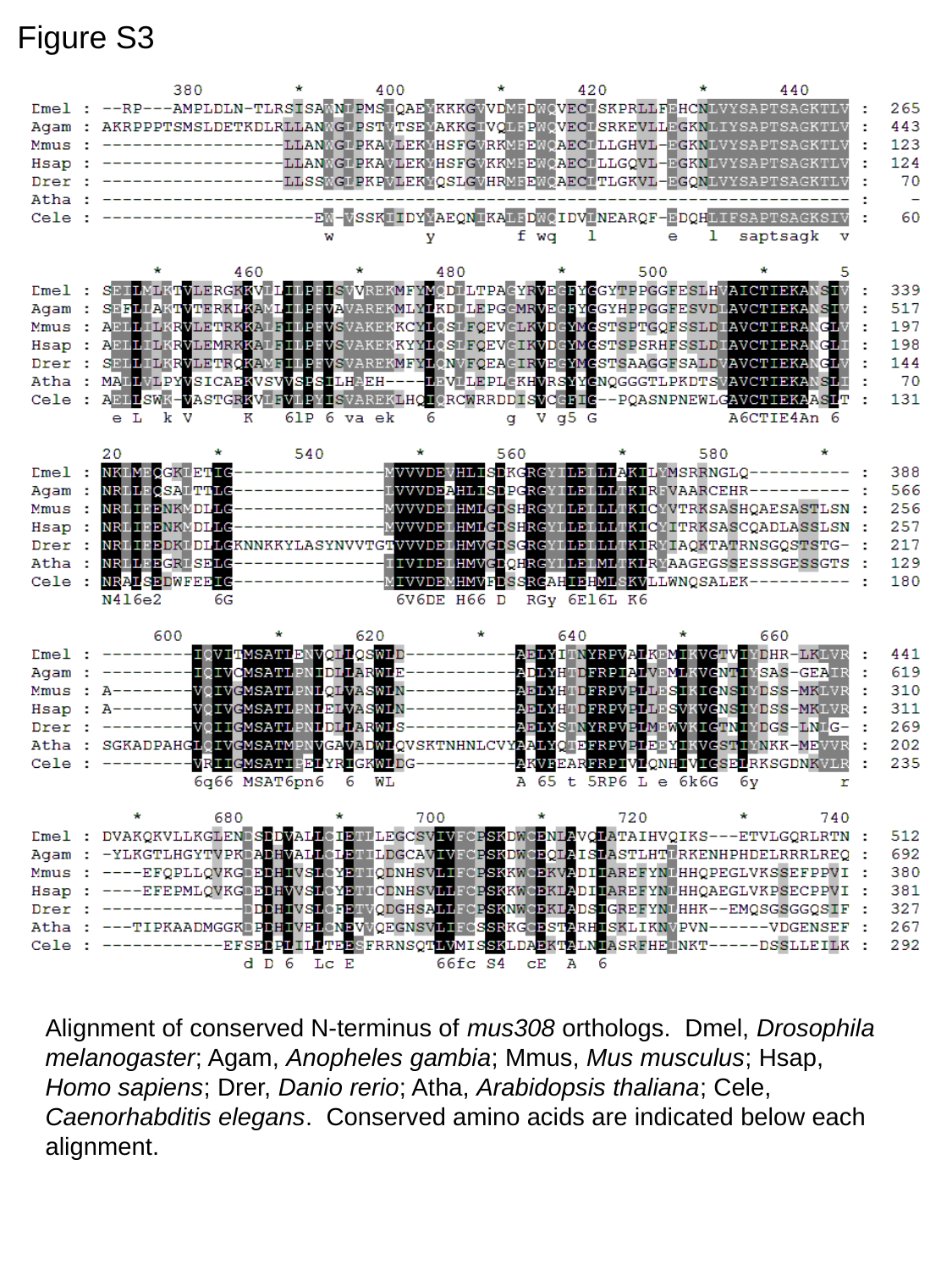

Figure S3
Alignment of conserved N-terminus of mus308 orthologs. Dmel, Drosophila
melanogaster; Agam, Anopheles gambia; Mmus, Mus musculus; Hsap,
Homo sapiens; Drer, Danio rerio; Atha, Arabidopsis thaliana; Cele,
Caenorhabditis elegans. Conserved amino acids are indicated below each
alignment.

## Slide 2
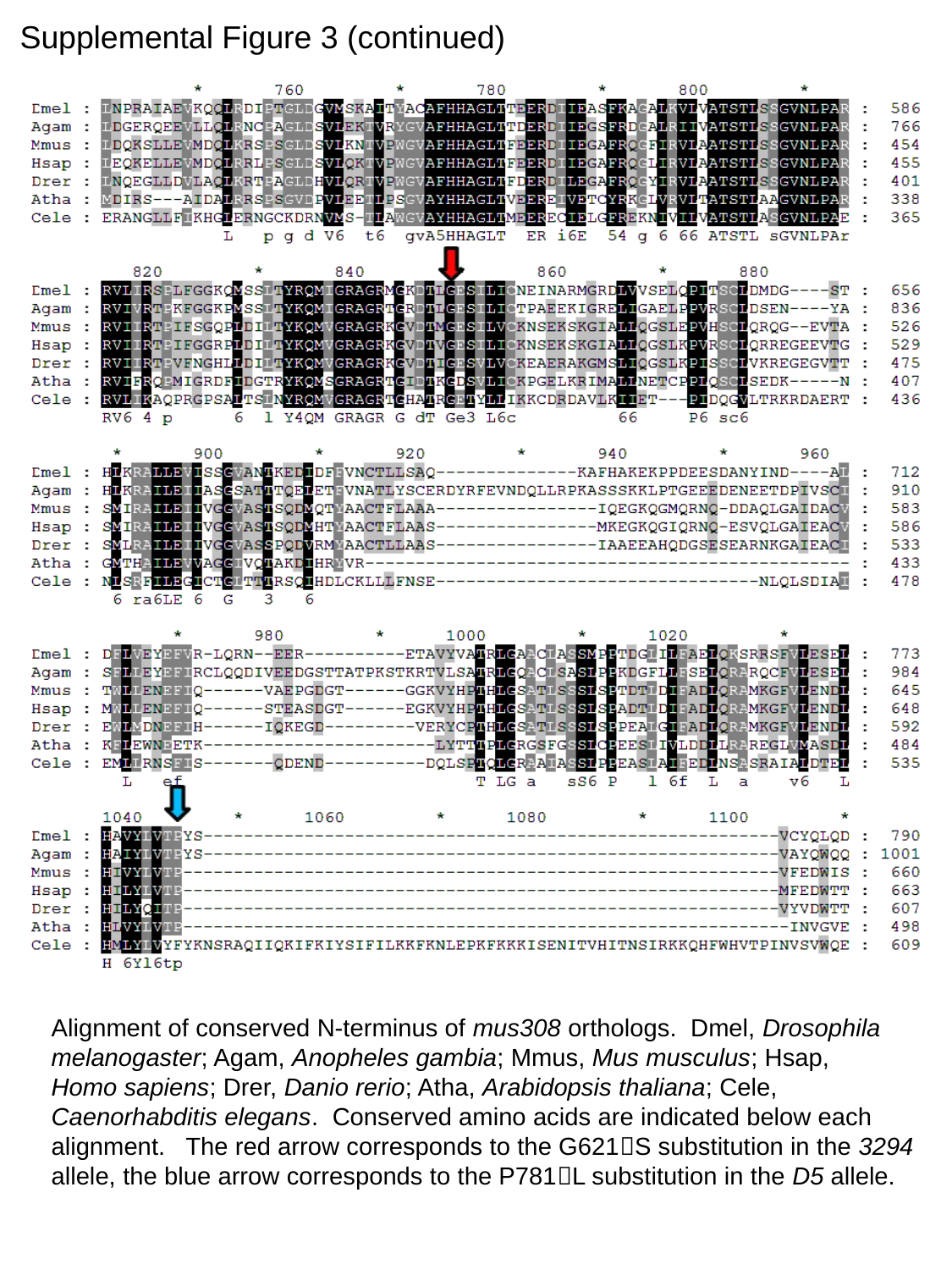

Supplemental Figure 3 (continued)
Alignment of conserved N-terminus of mus308 orthologs. Dmel, Drosophila
melanogaster; Agam, Anopheles gambia; Mmus, Mus musculus; Hsap,
Homo sapiens; Drer, Danio rerio; Atha, Arabidopsis thaliana; Cele,
Caenorhabditis elegans. Conserved amino acids are indicated below each
alignment. The red arrow corresponds to the G621S substitution in the 3294
allele, the blue arrow corresponds to the P781L substitution in the D5 allele.

## Slide 3
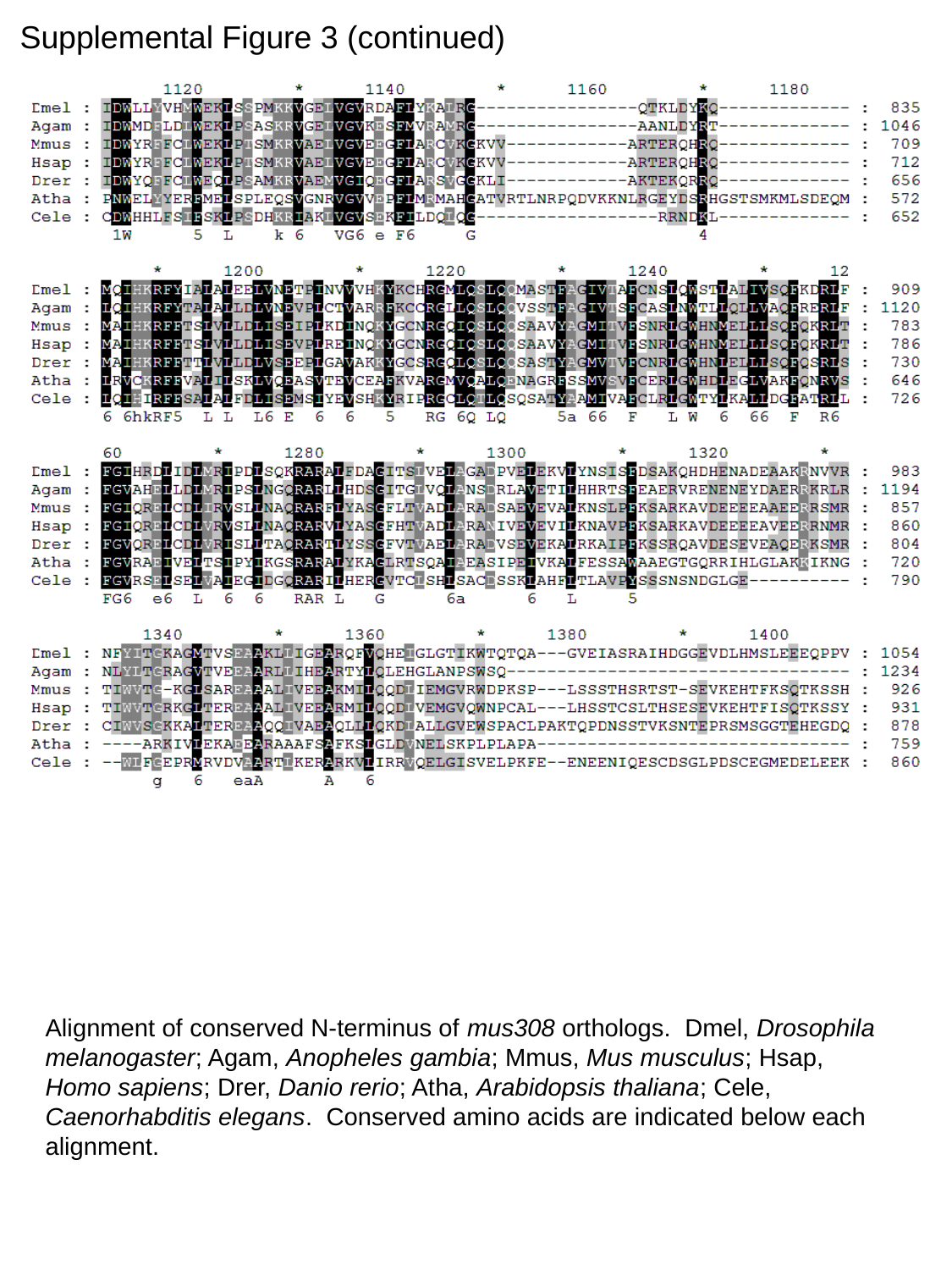

Supplemental Figure 3 (continued)
Alignment of conserved N-terminus of mus308 orthologs. Dmel, Drosophila
melanogaster; Agam, Anopheles gambia; Mmus, Mus musculus; Hsap,
Homo sapiens; Drer, Danio rerio; Atha, Arabidopsis thaliana; Cele,
Caenorhabditis elegans. Conserved amino acids are indicated below each
alignment.
